# Diagnosis of triple negative breast cancer using expression data with several machine learning tools

**DOI:** 10.6026/97320630018325

**Published:** 2022-04-30

**Authors:** Sankaranarayanan Pranaya, PK Ragunath, P Venkatesan

**Affiliations:** 1 Department of Bioinformatics, Sri Ramachandra Institute of Higher Education and Research, Porur, Chennai - 600 116, India; 2 Department of Statistics, ICMR, National Institute for Research in Tuberculosis, Chetpet, Chennai - 600 031, India

**Keywords:** Triple Negative Breast Cancer (TNBC), Breast Cancer, Machine learning, Artificial Neural Network (ANN), Logistic Regression, Radial Basis Function (RBF), Multi-Layer Perceptron (MLP)

## Abstract

Breast cancer is one of the top three commonly caused cancers worldwide. Triple Negative Breast Cancer (TNBC), a subtype of breast cancer, lacks expression of the oestrogen receptor, progesterone receptor, and HER2. This makes the prognosis poor and early detection hard. Therefore, AI based neural models such as Binary Logistic Regression, Multi-Layer Perceptron and Radial Basis Functions were used for differential diagnosis of normal samples and TNBC samples collected from signal intensity data of microarray experiment. Genes that were significantly upregulated in TNBC were compared with healthy controls. The MLP model classified TNBC and normal cells with anaccuracy of 93.4%. However, RBF gave 74% accuracy and binary Logistic Regression model showed an accuracy of 90.0% in identifying TNBC cases.

## Background:

Breast cancer is a highly heterogeneous disease and accumulation of distinct malignancies that expresses in the mammary glands. Carcinomas make up the common of breast cancers while sarcomas such as phyllodes tumors and angio-sarcomas are rarely seen. Providing an accurate prognostication for breast cancer patients is significant in order to inform them exactly about the course of the disease and to assign them to the right treatment modality [1]. It is the most common cancer in women worldwide. In 2020, there were 2.3 million women diagnosed with breast cancer and 685,000 deaths globally. As of 2020, there were 7.8 million women alive diagnosed with breast cancer in the past 5 years, making it the world's most prevalent cancer. Even if all of the potentially modifiable risk factors could be controlled, this would only reduce the risk of developing breast cancer by at most 30% [2]. Most common types of breast cancer are invasive ductal carcinoma and invasive lobular carcinoma. Presence of certain inherited high penetrance genes like BRCA1, BRCA2, BARD1, TP53, PALB2 mutations might be a cause for increased risk. Triple-Negative Breast Cancers (TNBCs), a breast tumor type defined by lack of estrogen receptor, progesterone receptor and human epidermal growth factor receptor 2 (HER-2) accounts for about 10-15% of the total breast cancer. Most of the epidemiological data shows that TNBC frequently occurs in premenopausal young women under 40 years old, which is approximately 15-20% of patients who have history of breast cancer [3]. Associated with other breast cancer subtypes, the mortality of TNBC patients is quite higher (>40%) within the first 5 years after diagnosis [4]. TNBC is extremely invasive, and patients may have reserved metastasis. The average duration for survival after metastasis is only 13.3 months, and the relapse rate is above 25% after the surgery. Reserved metastasis mostly involves the visceral organ and brain and generally occurs in the 3rd year after diagnosis [5]. In TNBC, the molecular subtypes are luminal A (ER/PR+, HER2- Ki67 + < 20%, with the percentage representing the immunohistochemical staining results for patient samples), luminal B (ER/PR+, HER2 overexpression), HER2 overexpression (ER-, PR-, HER2 overexpression), basal-like TNBC (ER-, PR-, HER2-), and other special subtypes. Diagnosis is hard due to the absence of oestrogen, progesterone and HER2 receptors [6]. Classification of medical data is an important task in the prediction of any disease. The most common method to predict the condition is by measuring the expression levels of a large number of genes simultaneously or genotype multiple regions of a genome [7]. Microarray based gene expression profiling helps in better understanding of biologic heterogeneity of breast cancer. Breast cancer is now perceived as a heterogeneous group of different diseases characterized by distinct molecular aberrations, rather than one disease with varying histological features and clinical behavior [8]. Therefore, it is of interest to describe the diagnosis of triple negative breast cancer using expression data with machine learning tools.

## Methodology:

### Gene Expression data:

A comprehensive literature mining (Table 1 - see PDF) of all eligible studies on Breast cancer gene expression was carried out by searching Gene Expression Omnibus (GEO) datasets using the query given below: A1 AND (("B" AND ((H OR h)) A2 AND (("B" AND ((H OR h)) A3 AND (("B" AND ((H OR h)) Where, A1 = Gene Expression; A2= Expression array; A3= Microarray; B= Breast cancer; H= Homo sapiens; h=human The concept lexicon was limited to Homo sapiens so as to retrieve only datasets containing studies or data pertained to human beings.

### Gene expression profiling:

Gene expression profiling is a method to measure the expression levels of thousands of genes simultaneously and sometimes, even an entire genome. This can yield vital information on the functions and activities of the gene of our interest. Pre-processed datasets were chosen by systematic text mining technique as described above. GEO2R was used for the gene expression profiling analysis of the chosen dataset. Based on the mining, microarray datasets were retrieved from NCBI. GEO repository using accession number GSE45498 annotated in GPL16299 platform. The dataset comprises of 40 healthy normal samples, 160 with cancer, 54 meta-static samples. The gene expression profiling values were log (base2) transformed and percentage shift normalization was performed. The fold change differences in gene expression between normal and disease samples were calculated for each gene separately. A cut off value of 1.25 fold change was used to classify up regulated genes [9] (Table 2 - see PDF).

### Machine learning models for differential diagnosis for TNBC from normal samples:

Machine Learning is a branch of Artificial Intelligence (AI) that employs a variety of statistical, probabilistic and optimization techniques that allows computers to "learn" from past examples and to detect hard-to-discern patterns from large, noisy or complex data sets [10]. These statistical models are used to classify, predict, diagnose and analyze data to reduce false decisions. Binary logistic regression and Artificial Neural Network models - Multilayer Perceptron (MLP) and Radial Basis Function (RBF) were built for the purpose of accurately classifying TNBC samples from normal.

### Logistic regression:

Logistic regression is one of the Machine Learning algorithms, which comes under the Supervised Learning technique. It is a predictive analysis algorithm that predicts a categorical dependent data variable by analysing the independent variables that are present. The most commonly used model is binary logistic regression model. When dealing with multiple genetic factors and other covariates, logistic regression assumes a linear relationship among the predictors and uses a logit link to combine them into a one-dimensional fitted value [11].

### ANN Model:

Artificial neural networks (ANNs) are statistical models which are able to abstract patterns in the observed data without the need for moulds about the relationships between the numerous variables [12]. ANNs are designed to present a mapping between the input layer and output layer by accepting intrinsic relationships between data [13]. There are various types of ANNs; MLP and RBF were used in this study (Figure 2 - see PDF).

### Multilayer perception based Neural Network model:

MLP is the most utilized model in neural network applications using the backpropagation training algorithm [14]. An MLP uses dot products and sigmoidal activation functions (or other monotonic functions such as Rectified Linear Unit) and designed of neurons grouped in an input layer, several hidden layers and an output layer. A neuron is connected from a layer to all neurons in the next layer; though, there is no connection between neurons in one layer. Training method is usually done through back propagation for all layers An ANN can have a number of hidden layers; theoretical research undertaken in this field presented that any complex and nonlinear function could be approached by a hidden layer for these models [15]. This study has employed MLP with one hidden layer and hyperbolic tangent activation function.

### Radial basis function based Neural Network model:

RBF networks uses Euclidean distances and Gaussian activation functions, which makes neurons more locally complex. RBF has two layers; the first layer is radial basis and the output layer is linear. RBF has used SoftMax as an activation method. Training process is done by competitive learning or clustering. Network performance can be improved by changing these parameters. By applying inputs to the network, the distance between input vectors and weight vectors is calculated and vector product is obtained by multiplying the calculated values by bias values. Then, these values generate as many neurons as inputs by corresponding functions; finally, output values are obtained by output layer [16]. In this study, Machine learning model was built by taking the independent variables as input layers and dependent variables as output layers. The dependent layers are the 15 genes and the independent variables are normal and triple negative cancer genes.

### Evaluating the goodness of the ROC curve:

To predict the goodness of values by the developed MLP and RBF models, the Receiver Operating Curve (ROC) was employed. ROC is a graphical display of sensitivity (True positive results (TPR) on y-axis) and (1 - specificity) and (false positive results (FPR) on x-axis) for fluctuating cut-off points of tested values ranged from 0 to 1. The Area under the Curve (AUC) is an effective and combined measure of sensitivity and specificity for assessing inherent validity of a diagnostic test. Maximum AUC = 1 and it shows that the diagnostic test is perfect in differentiating disease with non-disease subjects. This infers both sensitivity and specificity are one and both errors - false positive and false negative–are zero. This can happen when the distribution of disease and non-disease test values does not overlap. This is extremely unlikely to happen in practice. The AUC closer to 1 indicates better performance of the test. The diagonal connecting the point (0, 0) to (1,1) divides the square into two equal parts and each has an area of 0.5. When ROC is this line, overall, there is 50-50 chances that test will correctly discriminate the disease and non-disease subjects. The minimum value of AUC should be considered 0.5 instead of 0 because AUC = 0 means the test incorrectly classified all subjects with disease as negative and all non-disease subjects as positive. If the test results are reversed, then area = 0 is transformed to area = 1 (Figure 3 - see PDF).

## Results & Discussion:

Cancer treatment has progressed substantially over the past years with a reduction in therapy intensity, both for loco regional and systemic therapy; avoiding over treatment but also under treatment has become a major focus [17]. There is an absence of specific treatment strategies for this tumour subgroup, and hence TNBC is managed with conventional therapeutics, often leading to systemic relapse. Different molecular methods have been used to target TNBC, but the success rate is low. The current study is aimed at differential diagnosis of Triple Negative Breast Cancer and normal samples using microarray data, to build an Artificial Intelligence (AI) based model for diagnosing active disease with healthy control using differentially expressed genes based on signal intensity. Machine learning methods like Binary Logistic Regression, MLP and RBF can play a significant role in differential diagnosis of TNBC and normal healthy samples. Differential geneexpression profiling of the selected microarray datasets was carried out by using GEO2R and p-values were adjusted. Only those differentially regulated genes with p-value < 0.05 and top log 1.25 folds of upregulated genes were chosen for further analysis. The gene list is ESR1, IGFBP6, NGFR, DLC1, TGFBR3, EGR1, NTRK2, PPARG, CD34, IGF1, FOS, CAV1, FGF2, KIT and AR.

## Binary Logistic Regression:

After 13 iterations of Binary Logistic Regression, the genes EGR1 and PPARG were identified to be significant (p<0.05). Binary logistic regression model thus generated showed net accuracy of 90.0%. The derived logistic equation is as follows:

Y= EGR1 * (-0.03) + PPARG * (- 0.025) + 3.63

## Artificial Neural Network:

Upregulated 15 genes were taken as input layer and the category as output layer. All data were standardized, 70% of data was allocated as training set and the remaining 30% as test set. MLP was built with one hidden layer, the resultant neural network was found to be capable of classifying TNBC cases with 94.9% accuracy and controls with 88.2% of accuracy. The overall accuracy of MLP was found to be 93.4% (Table 3 - see PDF) Radial basis function network was found to be capable of classifying TNBC cases with 97.1%accuracy and controls with 20% of accuracy and it was found to be capable of classifying with an accuracy of 74.0%. MLP was found to perform better than RBF and binary logistic regression (Table 3 & 4 - see PDF). To compare two different classification models, AUC was calculated (Normal-0.924; TNBC-0.924) for the two models and ROC was constructed. The high AUC value is connected to high precision rate. In ROC space X-axis is Specificity and Y-axis is sensitivity. At the standardized specific threshold, the model outputs specificity (94.9%) and sensitivity (93.4%), to draw a point in ROC space. All the point of Normal and Triple negative breast cancer joins into ROC curve. The ROC curve revealed highly significant classifying ability among the disease diagnosis [18]. The relationship between sensitivity and specificity to precision depends on the percentage of positive cases among the total number of the samples collected. Hence high precision means that more significant results than inappropriate ones. The ROC curves of the two models are shown in Figure 3(see PDF) with ROC curve of MLP and RBF. Cancer diagnosis using Multi-Layer Perceptron, Radial Basis Function Networks, Learning Vector Quantization and competitive Learning Networks is known [19-21]. Among 15 genes, 8 genes - IGFBP6, DLC1, TGFBR3, EGR1, PPARG, CD34, FOS and AR were found to have independent variable importance above 40%. This data provide additional insights to known information for cancer diagnosis.

## Conclusion:

We describe a novel machine learning model to differentially diagnose TNBC from normal samples. We have demonstrated the properties and advantages of the model using TNBC gene expression dataset. We have also presented the performance of Machine layer techniques such as Artificial Neural Network and regression analysis model. The performance of classification algorithms is usually examined by evaluating the accuracy of the classification. We used three machine learning models namely, MLP, RBF and Binary logistic regression model for the dataset. The overall classification accuracy has been presented in Table 5(see PDF). The MLP model shows an accuracy of 93.4% which produced better specificity and accuracy compared to the RBF (74% of accuracy) and the binary logistic regression model (90% of accuracy). Data shows that the order of effectiveness in diagnosis of different neural networks is MLP followed by RBF. Data also shows the strong ability of neural networks with better performance. These networks store prototypes that are looked up for deciding the network output. Data shows that good performance is given by MLP followed by RBF. These networks try to predict the decision boundaries in the form of curves. The higher performance of the MLP as compared to the RBF in the testing data is an indication of a higher generalizing capability of the MLP. RBF gave a reasonably poor performance for the dataset. This indicates the limitation of the recurrent architecture to solve the classification problems due to their highly localized nature. The efficacy of the machine learning models in different populations is to be validated.
